# Inactivation of Factor VIIa by Antithrombin *In Vitro, Ex Vivo* and *In Vivo:* Role of Tissue Factor and Endothelial Cell Protein C Receptor

**DOI:** 10.1371/journal.pone.0103505

**Published:** 2014-08-07

**Authors:** Rit Vatsyayan, Hema Kothari, Nigel Mackman, Usha R. Pendurthi, L. Vijaya Mohan Rao

**Affiliations:** 1 Department of Cellular and Molecular Biology, The University of Texas Health Science Center at Tyler, Tyler, Texas, United States of America; 2 Division of Hematology and Oncology, McAllister Heart Institute, Department of Medicine, University of North Carolina at Chapel Hill, Chapel Hill, North Carolina, United States of America; National Cerebral and Cardiovascular Center, Japan

## Abstract

Recent studies have suggested that antithrombin (AT) could act as a significant physiologic regulator of FVIIa. However, *in vitro* studies showed that AT could inhibit FVIIa effectively only when it was bound to tissue factor (TF). Circulating blood is known to contain only traces of TF, at best. FVIIa also binds endothelial cell protein C receptor (EPCR), but the role of EPCR on FVIIa inactivation by AT is unknown. The present study was designed to investigate the role of TF and EPCR in inactivation of FVIIa by AT *in vivo.* Low human TF mice (low TF, ∼1% expression of the mouse TF level) and high human TF mice (HTF, ∼100% of the mouse TF level) were injected with human rFVIIa (120 µg kg^−1^ body weight) via the tail vein. At varying time intervals following rFVIIa administration, blood was collected to measure FVIIa-AT complex and rFVIIa antigen levels in the plasma. Despite the large difference in TF expression in the mice, HTF mice generated only 40–50% more of FVIIa-AT complex as compared to low TF mice. Increasing the concentration of TF *in vivo* in HTF mice by LPS injection increased the levels of FVIIa-AT complexes by about 25%. No significant differences were found in FVIIa-AT levels among wild-type, EPCR-deficient, and EPCR-overexpressing mice. The levels of FVIIa-AT complex formed *in vitro* and *ex vivo* were much lower than that was found *in vivo.* In summary, our results suggest that traces of TF that may be present in circulating blood or extravascular TF that is transiently exposed during normal vessel damage contributes to inactivation of FVIIa by AT in circulation. However, TF’s role in AT inactivation of FVIIa appears to be minor and other factor(s) present in plasma, on blood cells or vascular endothelium may play a predominant role in this process.

## Introduction

Tissue factor pathway inhibitor (TFPI) is the primary physiological regulator of factor VIIa (FVIIa)-tissue factor (TF)-induced blood coagulation [1], [2]. Although antithrombin III (AT) was shown to inhibit FVIIa [3]–[6], the physiological significance of this inhibition was debatable [7], [8]. AT could effectively inhibit FVIIa only when it was bound to TF and not free FVIIa [3], [4]. Still, compared to TFPI, AT was a poor inhibitor of FVIIa-TF [5], [8], [9]. Interestingly, Smith et al. [10] showed that levels of FVIIa-AT complex were surprisingly abundant in plasma (2% of plasma FVII antigen), and suggested that AT could be a significant regulator of FVIIa function and turnover in plasma. Recently, Agerso et al. [11] showed that rFVIIa-AT complex formation was responsible for 65% of the total rFVIIa clotting activity clearance following intravenous administration of rFVIIa in hemophilia patients. This is somewhat surprising as *in vitro* studies showed little inhibition of FVIIa by AT in the absence of TF, even in the presence of saturating concentrations of heparin [3], [4]. Furthermore, circulating blood contains either no detectable TF, or at best, traces of TF [12]–[14]. The above studies raise an interesting question that whether TF, either circulating or intravascular, or some other factors in blood are responsible for relatively rapid inactivation of FVIIa by AT *in vivo.*

Since AT preferentially inhibits FVIIa bound to TF, and FVIIa-AT complex rapidly disassociates from TF [9], the circulating levels of FVIIa-AT complex is thought to be an important indirect indicator of intravascular TF exposure *in vivo* [10]. Therefore, a number of recent studies measured plasma FVIIa-AT levels in various patient groups to investigate whether FVIIa-AT levels in plasma could predict hypercoagulable state and thrombotic risk [15]–[18]. Although these studies indicate that the FVIIa-AT levels may be useful in identifying hypercoagulable state in specific patient groups, they strongly suggest that large prospective cohort studies were needed to consider the clinical application of FVIIa-AT complex determination [17]. More importantly, to date, there is no empirical data in the literature showing that exposure of blood to intravascular TF is primarily responsible for the generation of FVIIa-AT complex *in vivo*.

Studies from our laboratory [19] and others [20], [21] have established that FVIIa binds endothelial protein C receptor (EPCR) in a true ligand manner. The interaction between FVIIa and EPCR is capable of not only eliciting protease activated receptor-1 (PAR1)-mediated barrier protective signaling [22], [23], but also promotes internalization of the receptor–ligand complex [24]. At present, it is unknown whether FVIIa binding to EPCR influences AT inactivation of FVIIa. Recently, we have shown that rFVIIa administered to mice intravenously (i.v) associates with EPCR, and EPCR facilitates the entry of FVIIa from circulation into perivascular tissues [25]. Once entered into perivascular tissues, FVIIa was retained there in functionally active state for extended time periods (24 h to 7 days) [25], which sharply contrasts to the short circulating half-life (<30 min) of FVIIa [26]. Since EPCR is present primarily on the endothelium whereas TF is mainly localized in extravascular cells, it raises the possibility that TF may be involved in FVIIa retention in extravascular tissues.

Development of transgenic mice that express either low levels of human TF (low TF) or high levels of human TF (HTF) in place of murine TF were useful in obtaining valuable insights into TF’s role in hemostasis, thrombosis and vascular development [27], [28]. Compared to wild-type TF, low TF mice express ∼1% of TF [29] whereas HTF mice express ∼100% of TF, except in the heart [30]. Similarly, development of EPCR-deficient and EPCR-overexpressing mice helped in elucidating the role of EPCR in hemostasis and inflammation [31], [32]. In the present study, we used the above mice to investigate the role of TF and EPCR in generation of FVIIa-AT complex *in vivo*. In order to compare the rates of AT inactivation of FVIIa *in* *vitro, ex vivo* and *in vivo,* human rFVIIa was administered to mice, or added to whole blood or plasma. Evaluation of the role of TF and EPCR in AT inactivation of exogenously administered rFVIIa is clinically relevant as AT was believed to be primarily responsible for rapid inactivation of therapeutically administered rFVIIa to hemophilic patients [11]. In addition, we also measured endogenous levels of FVIIa-AT complex in wild-type, TF and EPCR transgenic mice.

## Materials and Methods

### Ethics statement

Human participants: Blood from healthy donors was obtained following a written consent. Human subject research was approved by the Institutional Review Board at The University of Texas Health Science Center at Tyler.

Animals: All studies involving animals were conducted in accordance with the animal welfare guidelines set forth in the Guide for the Care and Use of Laboratory Animals and Department of Health and Human Services, and approved by the Institutional Animal Use and Care Committee of The University of Texas Health Science Center at Tyler, Tyler, TX (Animal Welfare Assurance Number A3589-01; Protocol Number: 530).

### Reagents

Human rFVIIa was obtained from Novo Nordisk A/S (Maaloev, Denmark). Mouse rFVIIa was provided by Mirella Ezban/Lars Petersen, Novo Nordisk (Denmark). Affinity purified polyclonal antibodies against human FVIIa were provided by the late Walter Kisiel (University of New Mexico, Albuquerque, NM, USA). Murine FVIIa antibodies were raised in-house by immunizing rabbits with recombinant mouse FVIIa. Antithrombin and sheep anti-AT antibodies, for both human and murine, were purchased from Haematologic Technologies, Inc (Essex Junction VT, USA). Human factor X was from Enzyme Research Laboratories (South Bend, IN, USA). Chromogenic substrate Chromogenix S-2765 was from DiaPharma (West Chester, OH, USA). Rat anti-mouse TF mAb (1H1) antibodies were provided by Daniel Kirchhofer, Genentech, CA, USA. Donkey anti-sheep biotinylated IgG was obtained from Thermo Scientific (Rockford, IL, USA). Lipopolysaccharide (LPS) from *Escherichia coli* 0111:B4 were from Sigma (St. Louis. MO, USA). Streptavidin alkaline phosphatase (ALP) and BluePhos microwell phosphatase substrate system were from KPL (Gaithersburg, MD, USA).

### Cells

Primary human umbilical vein endothelial cells (HUVEC), EBM-2 basal medium, and growth supplements were purchased from Lonza (Walkersville, MD, USA). Endothelial cells were cultured in EBM-2 basal medium supplemented with growth supplements, 1% penicillin/streptomycin, and 2% fetal bovine serum.

### Mice

The generation of low TF and HTF mice was described earlier [29], [30]. Breeding pairs of EPCR-deficient (EPCR-def) and EPCR-overexpressing (EPCR-OE) mice were obtained from Chuck Esmon (Oklahoma Medical Research Foundation, Oklahoma City, OK, USA), and their generation was described in earlier reports [31], [32]. Where available, littermate controls were used as wild-type mice. Otherwise, wild-type mice were obtained from Jackson Laboratory (Bar Harbor, ME, USA) or in-house breeding program. All mice were in C57BL/6J genetic background.

### Administration of rFVIIa to mice, collection of blood and tissues

Human rFVIIa was administered to mice via the tail vein at a dose of 120 µg kg^−1^ body weight in 100 µL of Tris-buffered saline (TBS, 50 mM Tris–HCl, 0.15 M NaCl, pH 7.5). At various time intervals, ranging from 5 min to 7 days after rFVIIa administration, blood was collected either via submandibular vein or by cardiac puncture at right ventricle if it was a terminal time point. Only two or three blood samples were obtained from each mouse. Blood was collected into 1/10 volume of 0.13 M sodium citrate anticoagulant unless otherwise specified. Mice were subsequently exsanguinated by severing the renal artery and perfused by flushing ice-cold saline containing 5 mM CaCl_2_+1 mM MgCl_2_ through the heart. Knee bone joints were excised, rinsed briefly with ice-cold saline containing 5 mM CaCl_2_+1 mM MgCl_2_, and then processed for measuring FVIIa activity and antigen levels. Briefly, excised knee bone joints were cut into fine pieces with a sharp razor blade, and added to TBS buffer containing EDTA (20 mM) (0.5 mL buffer per 100 mg tissue), freeze-thawed, vortexed, and centrifuged to obtain clear supernatant for measuring FVIIa antigen or activity levels.

*Ex vivo* studies were conducted using blood drawn into factor Xa inhibitor, rivaroxaban (100 µg/ml blood) as an anticoagulant. Plasma was separated by centrifugation at 6, 000×g for 15 min.

### Isolation of microparticles (MPs) from plasma, and TF activity assay

Microparticles from mouse plasma were isolated essentially as described earlier [33]. TF activity in MPs was measured by adding either mouse (in wild-type mice) or human FVIIa (in low TF and HTF mice) (10 nM), and human FX (175 nM), and measuring the amount of FXa generated at the end of 1 or 2 h activation period in a chromogenic assay. In some experiments, TF activity was measured both in the presence and absence of neutralizing TF antibodies to determine TF-specific coagulant activity.

### Generation and isolation of cell-derived microparticles

Confluent monolayers of HUVEC cultured in 100 mm dish were infected with control and TF adenovirus [34] (10 moi/cell). Two days post-infection, cells were washed with buffer A (10 mM Hepes, 0.15 M NaCl, 4 mM KCl, 11 mM glucose, pH 7.5), and then treated with calcium ionomycin (10 µM) in buffer B (buffer A containing 5 mM CaCl_2_ and 1 mg/ml bovine serum albumin) for 20 min at 37°C. Supernatant was collected and centrifuged first at 200×g for 5 min to remove any cell debris, and then centrifuged for 1 h at 20,000×g at 4°C to sediment microparticles. Microparticle pellet was washed once with buffer A, resuspended in 100 µl of buffer A, and stored at 4°C until used. Characterization of microparticles derived from endothelial cells transfected with control adenovirus (TF^−^ microparticles) and TF adenovirus (TF^+^ microparticles) in prothrombin activation assay revealed that their prothrombin activation potential was essentially equal, indicating that both TF^−^ and TF^+^ MPs express equal amounts of phosphatidylserine. As expected, TF^+^ microparticles and not TF^−^ microparticles effectively activated FX. TF concentration in TF^+^ microparticles was estimated in factor X activation assay using known concentrations of relipidated TF as a standard.

### FVIIa antigen and activity assays

rFVIIa antigen levels were determined by ELISA using rabbit anti-human FVIIa as the capture antibody and biotinylated rabbit anti-human FVIIa as the detecting antibody. FVIIa clotting activity was measured in a FVIIa-specific clotting assay as described previously [25] using STart coagulation analyzer (Diagnostica Stago, Parsippany, NJ, USA). Briefly, 50 µl of sample was incubated with 50 µl of 100 nM soluble TF in 1 mM phospholipids (40% phosphatidyl choline/25% phosphatidyl serine/35% phosphatidyl ethanolamine) and 100 µl of FVII-deficient plasma (George King Biomedical Inc., Overland Park, KS, USA) for 3 min at 37°C, and clotting was initiated by the addition of 100 µl of 25 mM CaCl_2_. Varying known concentrations of rFVIIa (0.5 ng to 30 ng/ml for the ELISA; 0.25 to 50 ng/ml for the activity assay) were used to construct a standard curve.

### Measurement of FVIIa-AT complexes

Majority of experiments described in this study required the measurement of human rFVIIa-mouse AT complex. The amount of rFVIIa-AT complex generated in our experimental system was determined by using an in-house developed ELISA, which is specific to measure the complex between human rFVIIa and mouse AT. Briefly, rabbit anti-human FVIIa antibodies (5 µg/ml) were coated onto 96-well ELISA plate for overnight at 4°C. After blocking the wells with 0.1% gelatin, diluted samples were added to the wells to capture rFVIIa. After 1 h incubation at room temperature, unbound material was removed, wells were washed 4 times, and then sheep anti–mouse AT antibody (5 µg/ml) was added to the wells for 1 h, followed by donkey anti-sheep biotinylated IgG (1∶500), and streptavidin ALP (1∶200 dilution) for 1 h each with ample washings of the wells prior to the addition of each reagent. Color was developed by using BluePhos microwell phosphatase substrate system, and measured at 650 nm. A background reading obtained in a zero min time point sample was subtracted from the readings of other time points. To generate rFVIIa-AT standard, a known concentration of rFVIIa (100 nM, 5 µg/ml) was incubated with soluble TF (200 nM), mouse AT (1 µM), and heparin (10 U/ml) overnight at 37°C, and the samples were subjected to non-reducing SDS-PAGE followed by immunoblot analysis to evaluate the extent of FVIIa-AT complex formation. The concentration of rFVIIa-AT in the standard was determined by the relative band intensities of rFVIIa-AT and free rFVIIa. Typically, more than 80% of FVIIa was in complex with AT. Varying concentrations of rFVIIa-AT complex (0.325 to 80 ng/ml, FVIIa-AT concentration depicts ng FVIIa in complex with AT) were used to generate a standard curve. The lower detection limit of the assay was between 0.25 to 0.5 ng ml^−1^ rFVIIa-AT complexes. The assay was specific to detect human FVIIa-mouse AT complex (substitution of free human FVIIa, mouse FVIIa, mouse AT or mouse control plasma in place of human FVIIa-mouse AT complex gave a reading similar to that of the blank (buffer), OD<0.1).

A similar procedure was used for measuring mouse FVIIa- mouse AT complexes or human FVIIa-human AT complexes. For this, ELISA plates were coated with either rabbit anti-mouse FVIIa antibodies or anti-human FVIIa antibodies to capture FVII/FVIIa from that particular species. The species-specific FVIIa-AT complexes were detected using either sheep anti-mouse AT or anti-human AT. It may be pertinent to note here that anti-mouse FVIIa antibodies do not cross react with human FVIIa and *vice versa.* Mouse FVIIa-AT standard was made by complexing recombinant mouse rFVIIa with mouse AT, and human FVIIa-AT standard was made by incubating human rFVIIa with human AT as described in the above paragraph.

### Statistics

All *in vitro* and *ex vivo* experiments were repeated three or more times. For *in vivo* studies, 3 to 9 mice were used for each group. Analysis of a data set using statistical software (GraphPad, Prism vs. 4.03, La Jolla, CA, USA) where n was 8 or more passed the normality test (D’Agostino and Pearson omni bus normality test). The data were shown as the mean ± SEM. Statistical significance between the two experimental groups was determined by Students t-test. One-way analysis of variance was used to determine statistical significance among three groups.

## Results

### Formation of FVIIa-AT complex in buffer, plasma and blood

**Figure 1:**
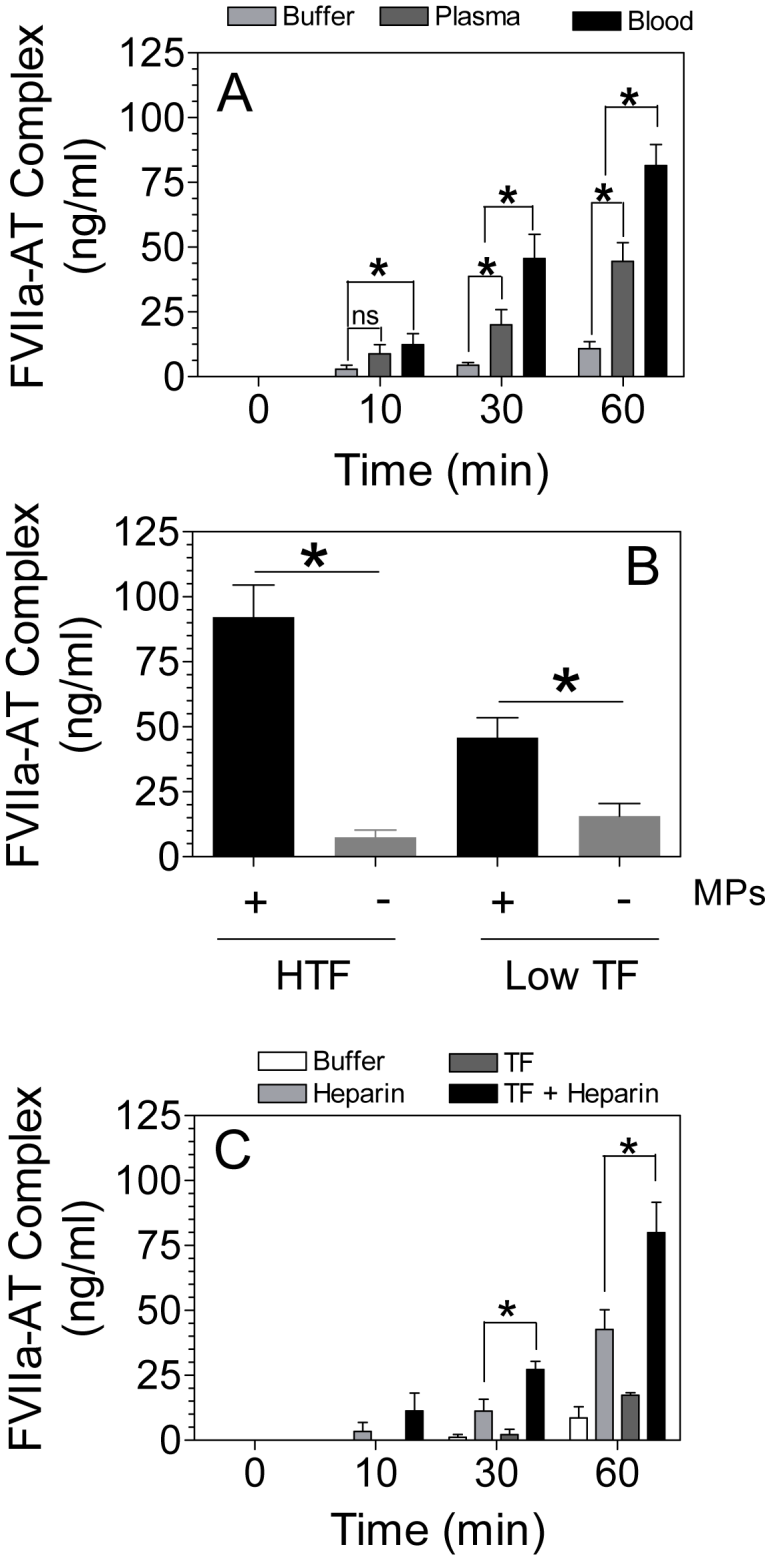
Comparison of FVIIa-AT complex formation *in vitro* and *ex vivo* conditions. (A) Blood from wild-type mice was drawn into rivaroxaban as an anticoagulant. Plasma was separated by centrifugation at 6,000×g for 5 min. To a reaction system not containing plasma or blood, a plasma concentration of AT (125 µg/ml) was added to Tris-buffered saline (TBS) containing 1 mg/ml bovine serum albumin (BSA), 5 mM CaCl_2_ and 1 mM MgCl_2_. rFVIIa was added to all three reaction systems (buffer, plasma and blood) to a final concentration of 1 µg/ml. At various time points, an aliquot was removed from the reaction mixtures and diluted 1∶10 in TBS/BSA containing 10 mM EDTA, and frozen immediately until they were used for the assay. Levels of FVIIa-AT complex was measured in an ELISA as described in Methods (n = 6 to 9). (B) Plasma obtained from HTF and low TF mice was divided into two equal aliquots, and one of the aliquots was subjected to centrifugation 20,000×g for 1 h at 4°C to remove microparticles. rFVIIa (1 µg/ml) was added to both the plasmas, and FVIIa-AT complex generated at 60 min was determined. (C) rFVIIa (1 µg/ml) was incubated for varying times with AT (125 µg/ml) in TBS/BSA buffer containing 5 mM CaCl_2_+1 mM MgCl_2_ in the presence or absence of heparin (1 U/ml) and relipidated TF (100 pg/ml). FVIIa-AT levels were determined in an ELISA (n = 4). The concentration of FVIIa-AT (ng/ml) reflects ng of FVIIa complexed with AT. *Indicates that the compared values differ in statistically significant manner (*P<0.05).*

First, we compared the rate of rFVIIa-AT complex formation in buffer, plasma, and blood in the absence of exogenously added heparin or TF. We included the plasma concentration of AT in a buffer system, and rFVIIa (1 µg/ml) was added to all three systems. As shown in Fig. 1A, increasing amounts of rFVIIa-AT complex was formed with increase in times under all experimental conditions. However, only less than 1% of FVIIa was found to be in complex with AT in a buffer system at the end of 1 h incubation with AT (Fig. 1A). The rate of rFVIIa-AT complex formation was significantly higher in plasma compared in buffer (about 4-fold). The rate of rFVIIa-AT complex formation was 2-fold higher in blood, compared in plasma.

It is possible that increased levels of rFVIIa-AT generated in plasma and blood, compared to a buffer system, may be due to the presence of traces of TF in plasma and blood. To investigate this possibility, we first attempted to measure TF antigen levels in mouse plasma. However, the sensitivity of TF ELISA assay was not suitable for detecting picograms of TF that may be present in plasma. Next, we measured MP-associated TF activity in plasma in factor X activation assay, using known concentrations of relipidated TF as the standard. This assay revealed that plasma of wild-type mice contains ∼2 pg/ml TF (data not shown). Actual concentration of TF in plasma may be slightly higher since this assay does not take into account soluble TF and TF that may not associate with MPs. To determine the role of trace amounts of TF in plasma in rFVIIa inactivation by AT, first we attempted to inhibit plasma TF activity by using TF neutralizing antibodies. However, both TF antibodies and control IgG interfered in measuring FVIIa-AT complex levels in an ELISA. Therefore, next we compared the generation of rFVIIa-AT complexes in plasma of low TF and HTF mice. As shown in Fig. 1B, the formation of rFVIIa-AT complex in plasma of HTF mice was significantly higher than in plasma of low TF mice. Furthermore, removal of MPs from the plasma by centrifugation significantly reduced the levels of rFVIIa-AT formation in plasma. These data indicate that traces of TF in plasma significantly accelerate inactivation of FVIIa by AT.

The effect of low concentrations of TF on FVIIa-AT complex formation was examined further in a buffer system where rFVIIa was incubated with AT ± heparin (1 U/mL) in the presence or absence of relipidated TF (100 pg/ml). As shown in Fig. 1C, a low concentration of TF substantially enhanced the formation of FVIIa-AT complex both in the absence and presence of heparin. In the presence of both TF and heparin, we observed ∼10-fold increase in the rate of FVIIa-AT generation in comparison to a buffer system containing AT alone. However, even in the presence of heparin and TF, only less than 10% of rFVIIa was found to be in complex with AT at the end of 60 min incubation time (Fig. 1C).

In additional studies, to examine the role of MPs on FVIIa-AT complex formation in plasma, endothelial cell-derived MPs bearing TF or lacking TF were added to human plasma or whole blood supplemented with rFVIIa (1 µg/ml), and the levels of FVIIa-AT formed at the end of 1 h was measured. The concentration of MP TF chosen for this study was 30 pg/ml, an outer limit of MP-TF concentration that could possibly be present in patients with high thrombotic risk (see rev [35] for references). Although we found a modest, 20 and 50%, increase in FVIIa-AT levels in plasma supplemented with TF^−^ and TF^+^ MPs, respectively, over the control plasma, this increase was not statistically significant (FVIIa-AT levels, ng/ml : no MPs, 6.14±1.45; TF^−^ MPs, 8.23±2.84; TF^+^ MPs, 11.13±5.9; n = 6). Heparin (1 U/ml) increased FVIIa-AT levels in plasma by 4-fold, but the presence of TF^−^ or TF^+^ MPs had no measurable influence on AT inactivation of FVIIa. Similarly, no differences were found in FVIIa-AT levels in whole blood in the presence or absence of MPs (data not shown).

### Role of TF in generation of FVIIa-AT complex *in vivo*

First to determine the extent of FVIIa-AT complex formed *in vivo* in mice under basal conditions and the role of TF in generation of FVIIa-AT, we measured mouse FVIIa-AT complexes in plasma obtained from wild-type, HTF and low TF mice. Both wild-type and HTF mice contained similar levels of mouse FVIIa-AT complex (wild-type, 6.93±2.47 ng/ml, n = 6; HTF, 7.66±2.40 ng/ml, n = 8) whereas FVIIa-AT levels were substantially low in low TF mice (1.21±0.19 ng/ml, n = 12).

**Figure 2:**
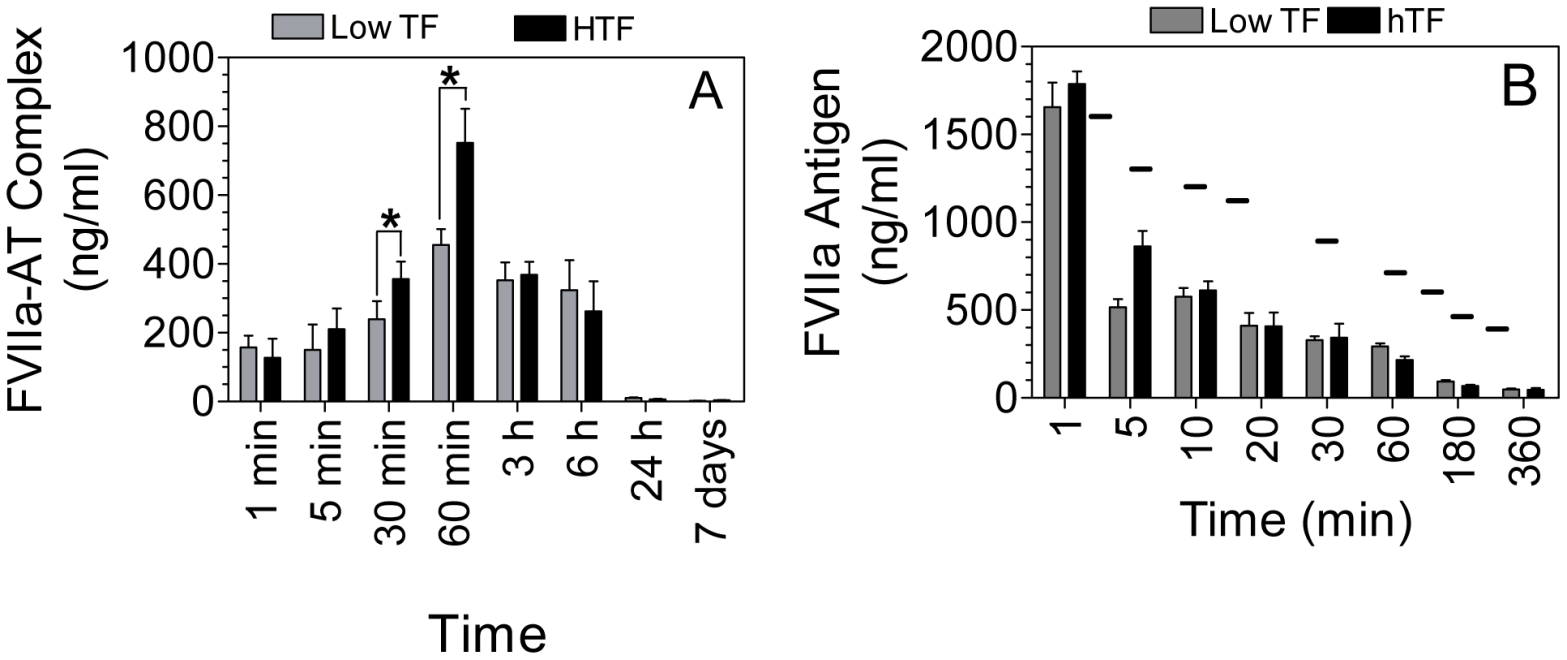
Levels of FVIIa-AT generated *in vivo* in HTF and low TF mice following administration of rFVIIa. HTF and low TF mice were injected with rFVIIa (120 µg/kg body weight) intravenously via the tail vein. FVIIa-AT complex (A) and FVIIa antigen (B) levels were determined in plasma obtained from these mice at various time points following rFVIIa administration (n = 4 or more). The small bar (−) in the figure represents expected FVIIa antigen levels, based on our previous study of rFVIIa clearance in wild-type mice using ^125^I-rFVIIa as a tracer [26], at 3, 5, 15, 30, 60, 120, 180, and 240 min following rFVIIa administration. The concentration of FVIIa-AT (ng/ml) reflects ng of FVIIa complexed with AT. *Indicates the value differs in statistically significant manner (*P<0.05)* between HTF and low TF mice at the given time point.

Next to compare the rate of AT inactivation of FVIIa *in vivo* vs. *in vitro* or *ex vivo*, and to determine the role of TF or other *in vivo* parameters in influencing AT inactivation of therapeutic concentrations of rFVIIa, low TF and HTF mice were administered with human rFVIIa (120 µg kg^−1^ body weight). Blood was collected at various time intervals following rFVIIa administration, and rFVIIa-AT complex and rFVIIa antigen levels in plasma were measured. As shown in Fig. 2A, rFVIIa complex formation with AT was evident as early as 1 min following rFVIIa administration in both the genotypes. rFVIIa-AT levels peaked at 60 min, and start to decline thereafter. At 24 h following rFVIIa administration, rFVIIa-AT complexes were barely detectable in plasma. The levels of rFVIIa-AT complex were significantly higher in HTF mice as compared with low TF mice at 30 and 60 min following rFVIIa administration (Fig. 2A). It may be pertinent to note here that measurement of TAT at these points showed no significant differences between HTF and low TF mice (TAT levels ng/ml at 30 min: low TF, 19.69±4.38; HTF 14.30±2.7; at 60 min: low TF, 24.27±9.78; HTF 18.93±4.7, n = 6).

We also measured rFVIIa antigen levels in the plasma of HTF and low TF mice to investigate whether TF influences pharmacokinetics of rFVIIa, which in turn could influence the formation of rFVIIa-AT complex *in vivo.* As shown in Fig. 2B, no significant differences were found in rFVIIa antigen levels in the HTF and low TF mice, indicating that differences in rFVIIa-AT complex levels in these mice at 30 and 60 min is not due to the difference in the plasma rFVIIa antigen levels in these mice.

The comparison of rFVIIa antigen and rFVIIa-AT complex levels, particularly in samples obtained at 60 min or later following rFVIIa administration, suggests that rFVIIa antigen assay does not fully recognize rFVIIa in rFVIIa-AT complex. Thus, rFVIIa antigen levels were substantially lower than rFVIIa-AT complex levels at these later time points, and also lower than predicted rFVIIa antigens levels based on our earlier FVIIa clearance studies utilizing ^125^I-labeled rFVIIa. Here, it may be pertinent to note that we evaluated several other FVIIa antibodies, both prepared in-house and obtained commercially or from other investigators, and none of the antibodies recognized rFVIIa in complex with AT to the same extent as of free rFVIIa.

### Role of EPCR in generation of FVIIa-AT complex *in vivo*

**Figure 3:**
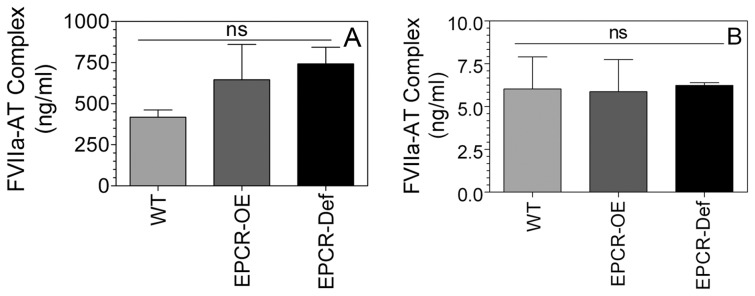
Levels of FVIIa-AT generated *in vivo* in wild-type, EPCR-overexpressing (EPCR-OE), and EPCR-deficient (EPCR-def) mice. (A) Wild-type, EPCR-OE and EPCR-def mice were injected with rFVIIa (120 µg/kg body weight) intravenously via the tail vein. FVIIa-AT levels in plasma obtained from these mice at 60 min post-rFVIIa administration was measured in an ELISA assay (n = 3–4). (B) Endogenous FVIIa-AT levels. Plasma obtained from wild-type, EPCR-OE and EPCR-def mice that were not subjected to any treatment were used to measure endogenous FVIIa-AT levels. The concentration of FVIIa-AT (ng/ml) reflects ng of FVIIa complexed with AT. ns, not statistically significant as determined in one-way analysis of variance.

Recently, we have shown that FVIIa binds EPCR both *in vitro* and *in vivo* [19], [36]. Therefore, we investigated here whether FVIIa binding to EPCR influences FVIIa-AT complex formation *in vivo.* Human rFVIIa was injected into wild-type, EPCR over-expressing (EPCR-OE), and EPCR-deficient mice (EPCR-def) and the levels of rFVIIa-AT complex were measured. As shown in Fig. 3A, there were no significant differences in the levels of rFVIIa-AT complex generated among the wild-type, EPCR-def and EPCR-OE mice. We also measured endogenous FVIIa-AT levels in the above groups of mice (no rFVIIa was administered). These data also showed no significant differences in FVIIa-AT levels among the three groups (Fig. 3B). It may pertinent to note here that while human FVIIa binds murine EPCR, murine FVIIa does not bind murine EPCR in any significant manner [36].

### Effect of enhanced TF expression on FVIIa-AT complex formation *in vivo*

**Figure 4:**
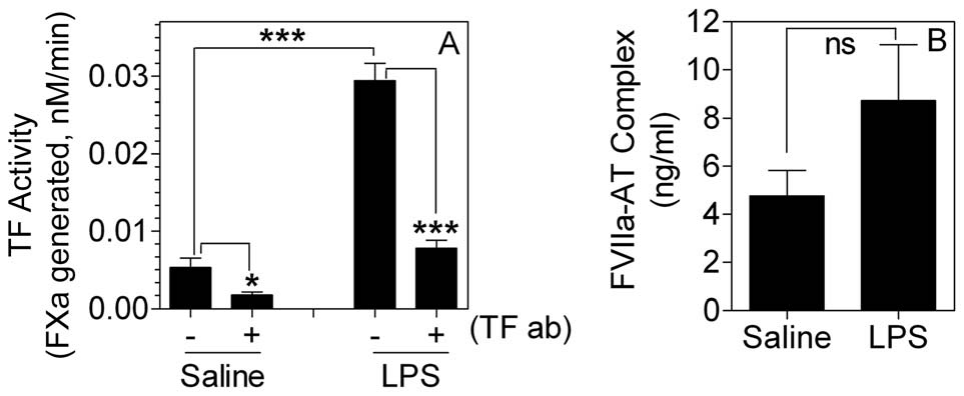
Measurement of microparticle TF procoagulant activity and FVIIa-AT levels in plasma following LPS administration in wild-type mice. Wild-type mice (C57BL/6J) were injected with saline or LPS (5 mg/kg body weight) intraperitoneally. 5 h after saline or LPS administration, blood was obtained from these mice by cardiac puncture, and plasma samples were processed for isolation of microparticles to measure TF procoagulant activity (A) or for determination of FVIIa-AT levels (B). Procoagulant activity measured in factor Xa generation assay in the absence or presence of mouse TF mAb (10 µg/ml, preincubated for 30 min) (n = 5). *Indicates the values differ in statistically significant manner between the control and LPS-treated mice. The concentration of FVIIa-AT (ng/ml) reflects ng of FVIIa complexed with AT. *, *P*<0.05; ***, P<0.001; ns, not statistically significant.

If traces of TF contribute to the generation of FVIIa-AT complex formation *in vivo,* then pathological conditions (e.g., sepsis, atherosclerosis etc.), where TF expression is upregulated in cells that come in contact with blood, may accelerate FVIIa-AT complex formation. Wang et al. [33] showed that TF MP levels were increased several fold in endotoxemic mice. To test the effect of increased expression of TF *in vivo* on FVIIa-AT generation, wild-type mice were injected with saline or LPS (5 mg/kg body weight), and 5 h after LPS administration blood was collected from these mice by cardiac puncture, and plasma was processed for measuring MPs procoagulant activity and mouse FVIIa-AT complexes. As shown in Fig. 4A, LPS administration markedly increased the procoagulant activity of plasma MPs. The increased procoagulant activity was due to the generation of TF^+^ MPs since the incubation of MPs with mouse TF mAb blocked the increased procoagulant activity. LPS administration increased the levels of FVIIa-AT complexes in plasma by about 50% compared to the saline control, but the difference is not statistically significant, probably due to a wider variation in FVIIa-AT levels in these mice. It may be important to note here that measuring endogenous murine FVIIa-AT complexes in LPS administered mice may not provide a complete picture of the role of TF on AT inactivation of FVIIa since FVIIa-AT complex formation is limited by the availability of FVIIa. LPS administration may not significantly increase FVIIa levels *in vivo* [37].

**Figure 5:**
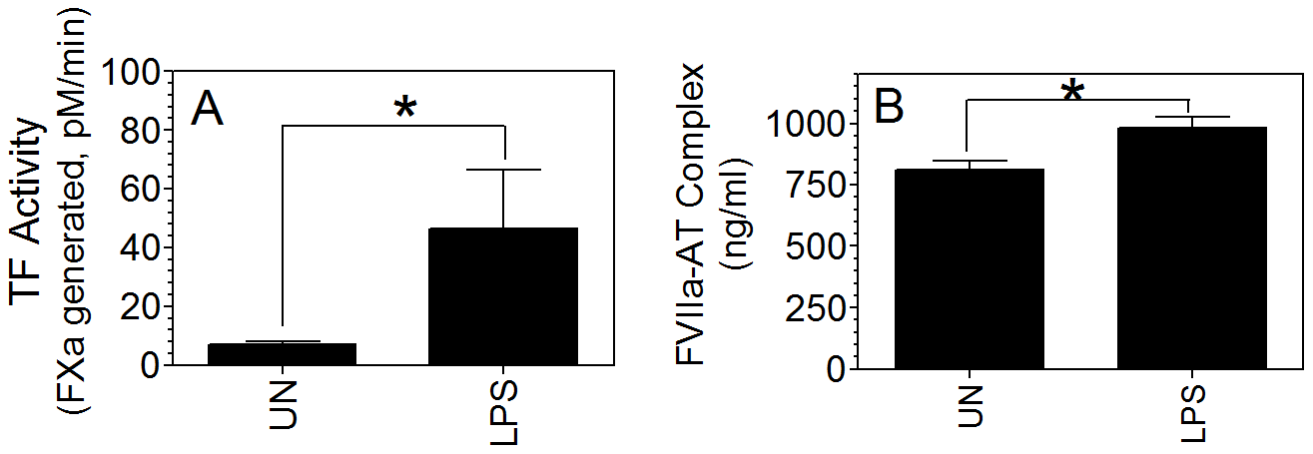
Effect of increased TF expression on formation of FVIIa-AT complex in HTF mice administered with therapeutic concentrations of rFVIIa. HTF mice were injected with LPS (5 mg/kg body weight) intraperitoneally. 5 h after LPS administration, rFVIIa (120 µg/kg body weight) was injected into these mice as well as unchallenged mice. 1 h following rFVIIa administration, blood was drawn from these mice by cardiac puncture, and plasma samples were processed for isolation of TF microparticles or determination of FVIIa-AT levels. (A) Microparticle TF activity; (B) FVIIa-AT levels (n = 4). The concentration of FVIIa-AT (ng/ml) reflects ng of FVIIa complexed with AT. *Indicates the values differ in statistically significant manner between the control and LPS-treated mice.

Therefore, to investigate the effect of enhanced *in vivo* TF on AT inactivation of FVIIa more accurately, HTF mice were injected with LPS (5 mg/kg body weight), and after 5 h, human rFVIIa was given to these mice via tail vein injection, and the amount of rFVIIa-AT complex generated in plasma was measured. As shown in Fig. 5A, MP TF activity was increased by 6-fold in HTF mice administered with LPS compared to control HTF mice. Measurement of rFVIIa-AT complex in these mice showed a slight but statistically significant increase in rFVIIa-AT levels in LPS-challenged mice compared to control mice (Fig. 5B). We also measured the levels of endogenous mouse FVIIa-AT complexes generated in the above experimental system. LPS treatment increased the levels of endogenous mouse FVIIa-AT in these mice by 40% (data not shown).

### Role of TF in retention of rFVIIa activity and antigen *in vivo*

**Figure 6:**
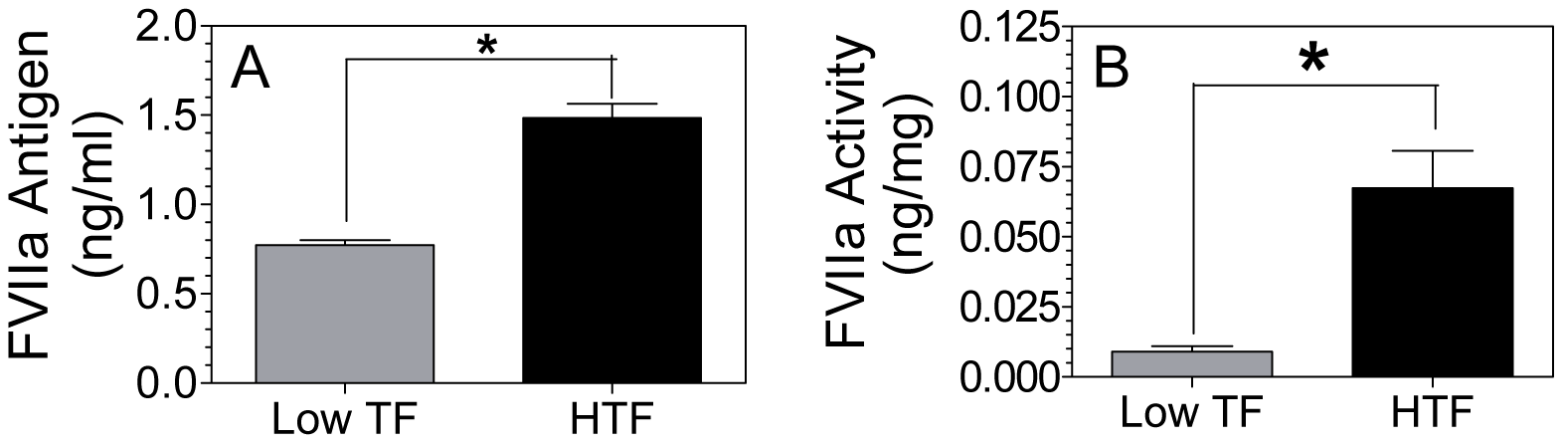
Higher levels of FVIIa retained in bone joints of HTF compared to low TF mice. HTF and low TF mice were injected intravenously via the tail vein with rFVIIa (120 µg/kg body weight). Seven days later, bone joints were collected, bone joint fluids were eluted, and assayed for FVIIa antigen (A) and FVIIa activity (B) levels (n = 3). *Indicates the value differs in statistically significant manner (*P<0.05)* between HTF and low TF mice.

Recently, we have shown that rFVIIa entered into extravascular tissues via EPCR-dependent mechanism is retained in tissues for extended time periods [25]. It has been thought that TF present on extravascular cells may play a role in retention of FVIIa in tissues [25]. Our earlier analysis of TF expression by immunohistochemistry in various tissues of wild-type mice revealed the presence of TF in bone joints in the zone of calcified cartilage in the growth plate region and the mineralized bone [38]. To investigate the effect of TF in retaining FVIIa in bone joints, rFVIIa was administered to low TF and HTF mice, and seven days after rFVIIa administration, bone joints from these mice were collected, and rFVIIa antigen and activity levels in bone joints were measured. As shown in Fig. 6A, rFVIIa antigen levels were significantly higher in the bone joints of HTF mice as compared with low TF mice. Measurement of FVIIa activity in these samples showed that rFVIIa in the bone joint of HTF is functionally active. As with rFVIIa antigen level, FVIIa activity level was substantially higher in bone joints of HTF as compared to low TF mice (Fig. 6B).

## Discussion

The present study carried out with low TF mice expressing ∼1% of wild-type TF and HTF mice expressing ∼100% of wild-type TF indicates that TF contributes to the formation of FVIIa-AT complex *in vivo.* Increased levels of FVIIa-AT complex were found in plasma of HTF mice compared to low TF mice endogenously as well as after administration of a therapeutic concentration of rFVIIa. Further, the observation that increased intravascular exposure of TF by LPS administration to HTF mice moderately, but statistically significant fashion, increased FVIIa-AT levels in the plasma confirms that increased FVIIa-AT levels in plasma may reflect exposure of blood to increased levels of intravascular TF. Nonetheless, our data also indicate that the effect of TF on AT inactivation of FVIIa appears to be modest, and therefore, measurement of FVIIa-AT levels in plasma may not be a very sensitive indicator of intravascular TF exposure, and may lack robustness in accurately predicting hypercoagulable state. For example, despite a ∼100-fold difference in TF expression between HTF and low TF mice, differences in FVIIa-AT levels between the two genotypes following rFVIIa administration was less than 2-fold, that too only at 30 and 60 min following rFVIIa administration but not at later time points. Similarly, LPS administration increased MP-associated TF activity in plasma of HTF mice by more than 600% whereas it only led to a small increase (∼25%) in FVIIa-AT levels in plasma.

Recent studies of pharmacokinetics of rFVIIa in hemophilia patients or animals showed that the majority of rFVIIa clotting activity following rFVIIa intravenous administration was inhibited by AT by forming rFVIIa-AT complexes [11], [39], [40]. These results suggest that AT inhibition of rFVIIa controls drug duration in hemophilia treatment with rFVIIa. Furthermore, these data also showed that the rate of inhibition of rFVIIa by AT is significantly higher *in vivo* than *in vitro* [40]. Consistent with these data, we found that most of rFVIIa in circulation was in complex with AT at 60 min following rFVIIa administration as the levels of FVIIa-AT complex measured at this time were very similar to total FVIIa antigen levels in the circulation (compare Fig. 2A and 2B). Although TF contributes to the formation of rFVIIa-AT complex *in vivo,* it does not appear to influence the overall pharmacokinetics of rFVIIa as rFVIIa was cleared in a similar fashion in low TF and HTF mice. These data were also consistent with the earlier observation that showed clearance of FVIIa was unaffected by its inactivation with AT [41]. The observation that rFVIIa-AT complex formation was impeded, but not markedly impaired in low TF mice indicates that TF plays probably a minor role in FVIIa inactivation by AT *in vivo.* Overall, our data suggest that although TF contributes to FVIIa-AT generation *in vivo*, it may not be the principle player that regulate rFVIIa inactivation by AT *in vivo*.

Comparison of rFVIIa-AT complex formation *in vitro, ex vivo* and *in vivo* conditions show that only traces of rFVIIa was complexed with AT *in vitro* (in buffer) in the absence of heparin. Although rFVIIa-AT complex formation was higher in plasma and blood, relative to that in buffer, still only less than 10% of rFVIIa formed complex with AT even after 1 h of incubation time. A significantly higher level of rFVIIa-AT formation in blood, compared to plasma, indicates that blood cells may promote rFVIIa inactivation by AT. At present, blood cell types that are responsible for this are unknown. Addition of heparin and a low concentration of relipidated TF increased FVIIa-AT formation in a buffer system, reaching to comparable level obtained in the blood in *ex vivo* condition. At present, it is unclear whether heparin-like proteoglycans and/or TF on blood cells is responsible for increasing FVIIa-AT complex formation in blood. It may be pertinent to add here that exogenous addition of TF^+^ MPs to human slightly increased AT inactivation of FVIIa in *ex vivo*, but this increase was not statistically significant. It is possible that other clotting factors in blood or other components of blood may contribute for the increased FVIIa-AT generation in blood. Here, one should note that both clotting factors IX and X were shown to enhance the inhibition of FVIIa-TF by AT on a human bladder carcinoma cell line [42]. In contrast to *in vitro* and *ex vivo*, rFVIIa complex formation with AT was relatively rapid *in vivo.* Within one minute following rFVIIa administration, the formation of rFVIIa-AT complex was evident, and most of rFVIIa present in circulation at 60 min following rFVIIa administration was in complex with AT. It is unclear at present the exact mechanism(s) by which FVIIa was rapidly inactivated by AT *in vivo.*

Recently, we have shown that rFVIIa administered to mice rapidly associates with EPCR on vascular endothelium *in vivo* [25], [26], [36]. Therefore, it is entirely possible that FVIIa binding to EPCR could facilitate FVIIa inactivation by AT. However, studies conducted herein with EPCR-deficient mice and EPCR overexpressing mice (at least 8- or more fold higher EPCR expression over the wild-type [32]) clearly show that EPCR does not play a role in FVIIa-AT complex formation *in vivo* as we observed similar levels of rFVIIa-AT complex in these mice following rFVIIa administration.

Our recent studies showed that although most of the rFVIIa administered to mice was cleared rapidly from circulation with a half-life of less than 30 min, a small amount of rFVIIa enters into extravascular tissues, and this FVIIa was retained for extended time periods in tissues [25], [26], [38], [43]. Since most of the extravascular cells express TF, and FVIIa binds to TF with a high affinity, it had been thought that TF may be playing a role in retaining FVIIa entered into extravasculature [25]. Our present observation of increased levels of rFVIIa activity and antigen in bone joints of HTF mice compared to low TF mice even 7 days after rFVIIa administration clearly supports the above hypothesis that FVIIa entered into extravasculature associates with TF, and this association could retain FVIIa for extended time periods in extravascular tissues. The potential significance of this finding to hemostasis and hemophilia therapy with rFVIIa had been discussed earlier [25].

In summary, data presented in the manuscript show that TF contributes to FVIIa-AT complex formation *in vivo.* However, presence of traces of intravascular TF alone cannot explain the generation of high levels of FVIIa-AT complex rapidly *in vivo* following rFVIIa administration. EPCR does not influence FVIIa-AT complex formation *in vivo.* Finally, our data also show that TF plays a role in retaining FVIIa entered into extravasculature.
